# Auditory object identification and localisation training for hyperacusis: preliminary effects on sound tolerance and noise acceptance

**DOI:** 10.3389/fnhum.2026.1983358

**Published:** 2026-10-09

**Authors:** Sana Aljoj, Grant Donald Searchfield

**Affiliations:** 1 Department of Audiology, The University of Auckland, Auckland, New Zealand; 2 Eisdell Moore Centre, The University of Auckland, Auckland, New Zealand; 3 TrueSilence Therapeutics Inc, Atlanta, GA, United States

**Keywords:** acceptable noise level, auditory training, hyperacusis, noise sensitivity, sound tolerance, therapy

## Abstract

**Background:**

Hyperacusis is characterised by reduced tolerance to everyday sounds and can substantially affect quality of life. This study investigated whether an auditory training programme designed to enhance auditory attention, auditory object identification, and sound localisation would reduce hyperacusis.

**Methods:**

Sixteen adults with hyperacusis completed a two-week auditory training intervention delivered through the QCore™ Tinnitus Therapy Platform. Participants completed 20 min of training daily using game-based sound identification and localisation tasks. Outcome measures were obtained before and after the intervention and included Loudness Discomfort Levels (LDLs), Acceptable Noise Levels (ANLs), and Noise Sensitivity Questionnaire (NoiSeQ) scores. Pre- and post-intervention performance was compared to evaluate changes in sound tolerance, acceptance of background noise, and perceived noise sensitivity.

**Results:**

Following the intervention, participants demonstrated significant improvements across all outcome measures. LDLs increased, indicating greater tolerance of tones. ANLs decreased, reflecting improved acceptance of background noise during listening tasks. NoiSeQ scores also improved, suggesting a reduction in self-reported noise sensitivity. Collectively, these findings indicate enhanced auditory tolerance and reduced sensitivity to environmental sounds were associated with training.

**Conclusion:**

A two-week auditory training programme was associated with improvements in objective and subjective measures of hyperacusis. The findings support the hypothesis that targeting auditory attention and auditory scene analysis may enhance sound tolerance and listening comfort in individuals with hyperacusis. The training represents a promising adjunct to existing hyperacusis management approaches. Controlled studies with larger samples and longer follow-up periods are warranted to confirm these findings and investigate their underlying mechanisms.

## 1 Introduction

Hyperacusis is a disorder of decreased sound tolerance in which everyday environmental sounds are perceived as excessively loud, uncomfortable, or painful despite being well tolerated by most people (Tyler et al., 2014; Khalfa et al., 2004). The condition can substantially affect quality of life, leading to avoidance of sound, social withdrawal, emotional distress, and difficulty participating in routine daily activities. Although hyperacusis is increasingly recognised in clinical practise, its underlying mechanisms remain incompletely understood and evidence-based treatment options remain limited (Pienkowski et al., 2014).

Current theories suggest that hyperacusis involves more than peripheral auditory abnormalities. While increased central auditory gain following reduced auditory input is considered a possible mechanism, the involvement of higher-order auditory and cognitive processes are important factors in noise sensitivity (Pienkowski et al., 2014; Khalfa et al., 2004; Welch et al., 2022). Altered attention to sound, emotion and context analysis have all been proposed as contributing factors to the degree of sensitivity (Wagenaar et al., 2010; Welch et al., 2022).

Auditory scene analysis (ASA) refers to the ability of the auditory system to organise complex acoustic environments into distinct auditory objects and streams (Bregman, 1994). This process allows listeners to focus on sounds of interest while suppressing competing auditory input, such as following a conversation in a noisy room. ASA relies on both bottom-up acoustic cues and top-down attentional mechanisms. In parallel, auditory attention enables listeners to selectively allocate cognitive resources to relevant sounds while filtering less important auditory information. Deficits in these processes have been implicated in conditions characterised by sound intolerance and auditory hypersensitivity, including hyperacusis, autism spectrum disorder, and attention-related disorders (Wagenaar et al., 2010; Lin et al., 2017).

Acceptance of background noise has commonly been assessed using Acceptable Noise Levels [ANLs, (Nabelek et al., 2006)], a measure of the maximum amount of background noise an individual is willing to accept while listening to speech. Research suggests that ANLs are influenced primarily by central auditory and cognitive processes rather than peripheral hearing sensitivity (Harkrider and Tampas, 2006). Studies investigating the neural basis of ANLs have demonstrated associations with auditory attention, sensory gating, and other higher-order auditory mechanisms (Miller et al., 2018; Sheehan et al., 2016). ANLs have been shown to improve following auditory training, indicating that tolerance of background sound may be modifiable (Bees et al., 2019). One such intervention is the Auditory Object Identification and Localisation (AOIL) training programme designed to target auditory attention and auditory scene analysis through active listening, sound identification, and spatial localisation exercises (Bees et al., 2019; Searchfield et al., 2007). Initially developed for tinnitus management, AOIL training has been associated with improvements in tinnitus-related outcomes and attentional control (Searchfield et al., 2007). Subsequent studies in individuals without sound tolerance disorders demonstrated that AOIL training could significantly improve acceptance of background noise, as reflected by reductions in ANLs (Bees et al., 2019). These improvements were hypothesised to result from enhanced auditory attention and sound segregation abilities, although the specific mechanisms remain unclear.

Despite growing evidence supporting the use of auditory training for auditory rehabilitation, its application to hyperacusis has not previously been investigated. Existing hyperacusis treatments, including counselling, sound therapy, tinnitus retraining therapy, and cognitive behavioural therapy, often focus on gradual sound exposure and desensitisation (Pienkowski et al., 2014). While these approaches may improve tolerance to sound, they typically rely on passive listening. In contrast, auditory training requires active engagement with acoustic information (Moore and Amitay, 2007) and may therefore provide an alternative means of modifying auditory processing and attention-related factors associated with sound intolerance.

The present study was designed as a preliminary evaluation of a novel auditory intervention for individuals with hyperacusis. The objective was not to determine the neurophysiological mechanisms underlying treatment effects, nor to provide definitive evidence of efficacy through a controlled clinical trial. Rather, the study sought to establish a proof of concept by examining whether participation in Auditory Object Identification and Localisation (AOIL) training was associated with measurable improvements in sound-tolerance outcomes in a clinical hyperacusis population. Demonstrating such changes in a preliminary cohort is an important first step in determining whether more extensive investigations, including controlled trials, mechanistic studies, and subgroup analyses, are warranted. The study evaluated whether the AOIL training programme could improve sound tolerance, acceptance of background noise, and perceived noise sensitivity in individuals with hyperacusis. Outcomes were assessed using Loudness Discomfort Levels (LDLs) (Cox et al., 1997), ANLs (Nabelek et al., 2006), and the Noise Sensitivity Questionnaire (NoiSeQ) (Schutte et al., 2007). In addition, participant engagement with the therapy was evaluated. It was hypothesised that AOIL training would improve sound tolerance, increase acceptance of background noise, and reduce perceived noise sensitivity for individuals with hyperacusis.

## 2 Methods

### 2.1 Ethics approval

Ethical approval for this study was granted by the University of Auckland Health Research Ethics Committee (Reference No. AH28824). All participants provided written informed consent prior to participation.

### 2.2 Participants

Participants were recruited through advertisements and a research volunteer database. Individuals who expressed interest in participating were provided with the Inventory of Hyperacusis Symptoms (IHS) (Greenberg and Carlos, 2018) questionnaire electronically. Candidates meeting the participation requirements were invited to attend an in-person assessment session at the University of Auckland. Each participant was assigned a unique identifier to ensure anonymised data collection and analysis.

### 2.3 Inclusion and exclusion criteria

Participants were eligible for inclusion if they were aged 18 years or older, reported either a diagnosis of hyperacusis or symptoms consistent with hyperacusis, obtained a score of 56 or greater on the IHS screening questionnaire, had no greater than a moderate hearing loss (air-conduction thresholds ≤55 dB HL), and owned a smartphone capable of downloading and running mobile applications. Individuals were excluded if they scored below 56 on the IHS, reported otologic conditions including cholesteatoma, otosclerosis, Ménière’s disease, auditory nerve lesions, or other significant auditory pathology, or had a medical condition that, in the opinion of the researcher, could place them at risk during participation.

### 2.4 Questionnaires and clinical measures

#### 2.4.1 Inventory of hyperacusis symptoms

The Inventory of Hyperacusis Symptoms [IHS, (Greenberg and Carlos, 2018)] was used as a screening measure of hyperacusis severity. The IHS is a 25-item questionnaire assessing the functional, communication, psychosocial, loudness, and emotional consequences of hyperacusis. Responses are rated on a four-point scale ranging from 1 (“not at all”) to 4 (“very much so”), yielding total scores between 25 and 100. Based on recommendations by Aazh et al. (2021), a score of 56 or greater was used as the eligibility criterion for participation. Scores of 80 or above were considered indicative of severe hyperacusis, while scores of 89 or above were considered representative of very severe hyperacusis.

#### 2.4.2 Hyperacusis history questionnaire

A Hyperacusis History Questionnaire, adapted from the University of California San Francisco Hyperacusis Patient Form^1^, was used to obtain demographic and clinical information. The questionnaire collected information regarding symptom onset, laterality, triggering sounds and situations, emotional and physical consequences, impact on daily life, hearing protection use, history of noise exposure, tinnitus, previous treatments, medication use, and general health status.

#### 2.4.3 Noise sensitivity questionnaire

The NoiSeQ, (Schutte et al., 2007) was administered before and after the intervention. The NoiSeQ contains 35 items assessing reactions to environmental noise across five domains: leisure, work, communication, habitation, and sleep. Each item is rated on a five-point Likert scale ranging from 1 (“strongly agree”) to 5 (“strongly disagree”). Composite scores are calculated across domains, with higher scores reflecting greater perceived sensitivity to noise. All questionnaires were administered electronically using REDCap (Research Electronic Data Capture).

### 2.5 Audiological assessment

Prior to audiometric testing, bilateral otoscopy was performed using a Welch Allyn™ otoscope to assess ear canal status and identify any contraindications to insert earphone use, including excessive cerumen, debris, discharge, or tympanic membrane abnormalities. Pure-tone audiometry was conducted in a sound-treated booth compliant with ISO 8253-1:2010 standards using a Grason-Stadler GSI AudioStar Pro™ audiometer. Air-conduction thresholds were obtained using ER-3A insert earphones. TDH-39/DD45 supra-aural headphones were used when cerumen prevented the use of insert earphones. Participants responded to stimuli using a handheld response button. Thresholds were measured using the modified Hughson-Westlake procedure (Carhart and Jerger, 1959) at 250 Hz, 500 Hz, 1 kHz, 2 kHz, 3 kHz, 4 kHz, 6 kHz, and 8 kHz. When tinnitus interfered with pure-tone detection, warble tones were substituted to improve response reliability. Bone-conduction thresholds were obtained using a RadioEar B81 bone vibrator at 500 Hz, 1 kHz, 2 kHz, and 4 kHz where clinically indicated. Participants meeting audiometric eligibility criteria proceeded to the remaining study procedures.

### 2.6 Loudness discomfort levels

LDLs were measured using the same audiometer and transducers employed for pure-tone audiometry. Testing was conducted bilaterally at 500 Hz, 1 kHz, 2 kHz, and 4 kHz. For participant safety, maximum presentation levels did not exceed 110 dB HL, or 105 dB HL at 500 Hz, corresponding to audiometer output limits. Participants were monitored continuously for signs of discomfort and were able to pause or discontinue testing at any time. Stimuli consisted of 1,000-ms pure tones presented with approximately 1,000-ms interstimulus intervals. Participants were provided with a seven-category loudness rating scale ranging from “very soft” to “uncomfortably loud.” The following standardised instructions, adapted from Cox et al. (1997), were provided: “I would like to determine the upper limit of loudness that you find comfortable. You will hear a series of tones that will sound soft at first and gradually increase in loudness. After each tone I would like you to point to the level on the chart that best describes how loud the sound appears to you.” Testing began at 10 dB above the participant’s hearing threshold for the corresponding frequency. Presentation levels were increased in 5-dB increments until the participant identified the sound as “loud but okay” (Category 6). The level was then reduced by 10 dB and increased again in 2-dB increments until Category 6 was selected a second time. This procedure was repeated three times at each frequency, and the mean of the three measurements was recorded as the LDL. Because the participants were sound-sensitive, the “loud but okay” criterion was used in place of “uncomfortably loud” to minimise participant distress while maintaining reliable pre- and post-intervention comparisons.

### 2.7 Acceptable noise levels

ANLs were obtained through measurements of Most Comfortable Level (MCL) and Background Noise Level (BNL). Testing was conducted in a sound-treated room compliant with ISO 8253-2:2009 standards. Speech stimuli consisted of the Arizona Travelogue passage [Cosmos Inc., (Nabelek et al., 2004)] with a male American speaker presented from an HP EliteBook 840 G5 laptop running Audacity software through an Analogue Connector 2-FastTrack Pro HS5 powered loudspeaker system. Two loudspeakers were positioned 130 cm from the participant at ±10° azimuth and separated by 50 cm. For MCL determination, the Arizona Travelogue was initially presented at 50 dB SPL, calibrated using a sound level metre at the listener’s ear. Participants were instructed to identify the level at which the speech was most comfortable by signalling “thumbs up” to increase the level and “thumbs down” to decrease it. Initial adjustments were made in 5-dB increments and subsequently refined using 2-dB steps until the participant identified their MCL. Following the determination of MCL, multitalker babble noise [revised SPIN test (Bilger et al., 1984)] was introduced at 30 dB SPL while the Arizona Travelogue continued at the participant’s MCL. Participants were instructed to adjust the background noise to the highest level they were willing to tolerate while continuing to follow the speech passage comfortably and without becoming tired or distressed. Background noise levels were initially adjusted in 5-dB steps and then refined in 2-dB increments until the participant’s BNL was established. ANL was calculated as ANL = MCL − BNL, with lower ANL values indicating greater acceptance of background noise.

### 2.8 Auditory training intervention

At the completion of the first assessment session, participants were onboarded to the QCore™ Tinnitus Therapy platform and provided with FW9 Stereo Open-Ear Wireless Earphones. The earphones were paired with participants’ smartphone, and a demonstration session was completed in the clinic to ensure familiarity with the software. The AOIL training consisted of two game-based auditory training activities designed to train auditory attention, auditory scene analysis, auditory object identification, and sound localisation. The first activity, *Sound Locator*, required participants to identify the spatial location of a target sound while ignoring competing sounds and environmental background audio. Participants were first presented with a target sound and instructed to remember and attend to it. They were then presented with one or more sounds originating from different spatial locations and were required to identify the location of the target sound from six possible options. Participants could replay sounds as often as required and were provided with up to five attempts per training round. Stimuli included environmental sounds such as ringing, buzzing, splashing, rattling, whooshing, and impact sounds, with durations ranging from one to 3 seconds.

The second activity, *Sound Identifier*, targeted both auditory attention and spatial localisation. During each trial, three environmental sounds were presented sequentially, with one designated as the target sound. Participants answered questions regarding either the location of the target sound or the identity of the sound occurring at a specified location. Example questions included “What did you hear on your right?” and “Where did you hear the metal impact?” Participants were permitted unlimited replay of each sound sequence but only one response attempt per question. Environmental sounds ranged from one to 6 seconds in duration.

#### 2.8.1 Training schedule

Participants were instructed to complete 20 min of Auditory Object Identification and Localisation (AOIL) training per day for 14 consecutive days. They could complete either training task exclusively or alternate between both tasks. The application included daily reminder notifications and displayed a timer indicating daily usage duration. Although game levels and scores were visible within the application interface, participants were informed that progression was unrelated to study outcomes. Training adherence was monitored remotely through a clinician dashboard that recorded daily application use.

### 2.9 Study procedure

Sixteen participants completed the study. Each participant attended two clinic sessions separated by a 14-day intervention period. The first session, lasting approximately 2 hours, included a review of study procedures and consent, completion of the Hyperacusis History Questionnaire and NoiSeQ, otoscopy and audiometric assessment, measurement of LDLs and ANLs, installation and demonstration of the auditory training application, and pairing and distribution of wireless earphones. Participants subsequently completed 20 min of AOIL training daily for 14 days. Following completion of the intervention period, participants returned for a second session lasting approximately 30–40 min. Assessments included repeat administration of the NoiSeQ, repeat LDL measurements, repeat ANL measurements, and a debriefing regarding participants’ experiences with the auditory training application.

### 2.10 Data collection and statistical analysis

Data were analysed using GraphPad Prism version 10.3.1 (GraphPad Software, San Diego, CA, United States). Normality was assessed using the Shapiro–Wilk test. As outcome variables were normally distributed, parametric statistical analyses were used throughout. Primary outcome measures were LDLs, ANLs, and NoiSeQ scores. Pre- and post-training measures were compared to determine the effects of the two-week smartphone-based AOIL training programme on sound tolerance, acceptance of background noise, and perceived noise sensitivity in individuals with hyperacusis. ANOVA was used and Tukey’s correction was applied to multiple paired *t*-tests.

## 3 Results

### 3.1 Participant characteristics

The sample comprised 12 females (75.0%) and 4 males (25.0%), aged 21 to 72 years (mean = 44.7 years, SD = 16.8). This study was designed as an exploratory pilot/case-series investigation. No *a priori* power analysis was conducted due to the absence of effect-size estimates for this intervention in hyperacusis populations. Participants reported co-morbidities including tinnitus (*n* = 11, 69%). None had previously received treatment for hyperacusis. The mean IHS score at screening was 73.2 (SD = 10.8), confirming clinically significant hyperacusis symptoms across all participants. Two participants met criteria for severe hyperacusis (IHS ≥ 80), and one participant met criteria for very severe hyperacusis (IHS ≥ 89). Hyperacusis had a substantial impact on daily functioning. Nine participants (56.3%) reported that normal conversational speech was excessively loud, and all participants indicated that emotional or physical stress exacerbated their symptoms. Hyperacusis interfered with sleep in nine participants (56.3%), exercise in eleven (68.8%), family activities in twelve (75.0%), and social activities in fourteen (87.5%). All participants reported that hyperacusis negatively affected work performance and concentration. Three participants reported a pre-existing hearing loss, including one participant who had previously used hearing aids. Mean audiometric thresholds were within normal limits across the speech frequencies, with a mild high-frequency decline bilaterally. Average right-ear thresholds were within normal hearing limits (≤15 dB HL) from 0.25–6 kHz, sloping to a slight hearing loss at 8 kHz. Average left-ear thresholds were normal from 0.25–3 kHz, with a similar high-frequency decline.

### 3.2 Training adherence

All participants completed the AOIL intervention. Participants completed an average of 11.8 training days (SD = 2.9) out of the prescribed 14 days. Mean training completion period was 14.5 days (SD = 1.6), with the second assessment occurring an average of 15.3 days (SD = 2.0) after training commenced and 0.8 days (SD = 1.4) following completion of training (Table 1). Participants aged 40 years and older demonstrated high completion rates, whereas younger participants aged 20–39 years showed lower completion rates.

**Table 1:** Mean completion rate (percent of prescribed training) of auditory training across the different age ranges.

Age range	Number of participants	Mean training completion rate %	SD	Min	Max
20–29	4	69.8	14.99	57	86
30–39	4	71.5	21.15	43	100
40–49	2	96.5	4.95	93	100
50–59	2	100	0	100	100
60–69	3	96.5	4.95	93	100
70–79	1	100	0	100	100

### 3.3 Loudness discomfort levels

Mean LDLs increased following completion of the auditory training programme across all tested frequencies and both ears (Figure 1).

**Figure 1:**
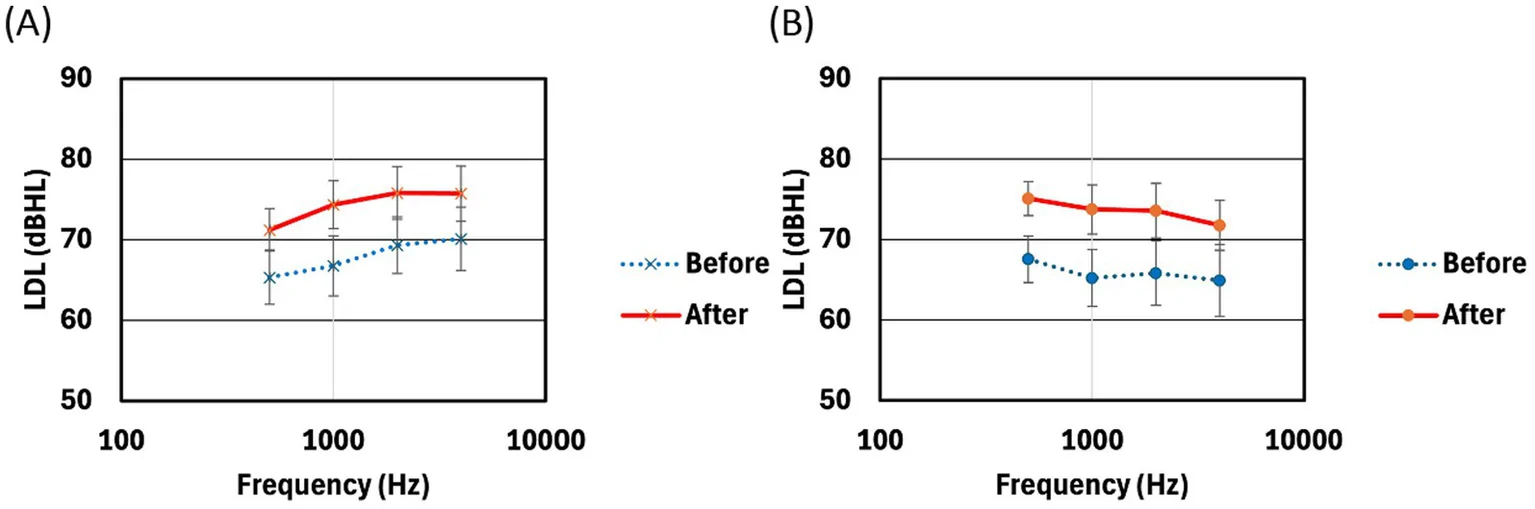
Average LDLs obtained before and after auditory training **(A)** Right ear. **(B)** Left ear. Error bars represent ±standard error of the mean.

To evaluate changes in loudness discomfort levels following AOIL training, LDL measurements obtained from both ears at 0.5, 1, 2, and 4 kHz were analysed using a two-way repeated-measures ANOVA with factors of Time (Before vs. After) and Ear/Frequency (8 LDL measurements). Because the assumption of sphericity was violated, Greenhouse–Geisser corrections were applied where appropriate.

The analysis revealed a highly significant main effect of Time, *F*(1, 15) = 35.88, *p* < 0.0001, indicating a significant overall increase in LDLs following training. Across all frequencies and ears, the mean LDL increased from 64.45 dB HL before training to 73.91 dB HL after training, representing an average improvement of 9.47 dB (95% CI: 6.10–12.83 dB). The Time effect accounted for 11.3% of the total variance, demonstrating a substantial improvement in loudness tolerance following the intervention.

There was no significant main effect of Ear/Frequency, *F*(2.43, 36.48) = 2.69, *p* = 0.07, indicating that LDLs did not differ systematically across frequencies and ears when averaged across time. Likewise, there was no significant Time × Ear/Frequency interaction, *F*(3.74, 56.11) = 0.62, *p* = 0.64, indicating that the magnitude of improvement following training was broadly similar across frequencies and ears. This finding suggests that AOIL training was associated with a generalised increase in sound tolerance rather than frequency-specific effects.

#### 3.3.1 *Post hoc* comparisons

Tukey-adjusted pairwise comparisons were performed separately for the pre-training and post-training assessments to examine differences among LDL measurements across frequencies and ears.

##### 3.3.1.1 Baseline (pre-training)

Prior to training, LDLs were generally similar across frequencies and ears. Of the 28 pairwise comparisons, only two remained statistically significant after correction for multiple comparisons: Left ear 4 kHz LDLs were significantly higher than right ear 4 kHz LDLs (mean difference = 6.46 dB, 95% CI 1.88–11.03 dB, *p* = 0.0034). Left ear 2 kHz LDLs were significantly higher than left ear 0.5 kHz LDLs (mean difference = 4.33 dB, 95% CI 0.38–8.28 dB, *p* = 0.0268). All other comparisons were non-significant (all *p* > 0.05), demonstrating relatively uniform loudness discomfort thresholds across frequencies and ears at baseline.

##### 3.3.1.2 Post-training

Following AOIL training, LDLs again remained broadly comparable across frequencies and ears. Only one significant *post hoc* comparison was identified: Left ear 2 kHz LDLs were significantly higher than right ear 4 kHz LDLs (mean difference = 4.02 dB, 95% CI 0.09–7.95 dB, *p* = 0.043). No other pairwise comparisons reached statistical significance after Tukey adjustment.

### 3.4 Acceptable noise levels

Mean MCL remained relatively stable following training (52.1 vs. 51.6 dB). In contrast, mean BNL increased from 33.4 dB to 39.1 dB, indicating greater tolerance of competing background noise. Consequently, mean ANL decreased from 18.7 dB before training to 12.6 dB after training, reflecting improved acceptance of background noise (Figure 2). Variability also decreased following training, as evidenced by lower standard deviations for BNL and ANL measurements. Paired-samples *t*-tests were used to compare ANL, MCL, and BNL measurements obtained before and after auditory training. A significant reduction in ANL was observed following training, *t*(15) = 4.21, *p* = 0.0008. Mean ANL decreased from 18.69 dB (SD = 5.67) at baseline to 12.56 dB (SD = 4.58) post-training, representing an average improvement of 6.13 dB (SD = 5.80). Participants, on average, demonstrated a 6.1 dB improvement in their ability to tolerate background noise after completing the training programme. The standard error of the mean difference was 1.46 dB, and the 95% confidence interval (CI) ranged from −9.23 dB to −3.03 dB. As this confidence interval does not include zero, the result provides robust evidence that the observed improvement was unlikely to be due to chance alone. The magnitude of the treatment effect was large. The partial eta-squared statistic (η^2^p = 0.54) indicates that approximately 54% of the variance in ANL scores was attributable to the pre-post intervention effect.

**Figure 2:**
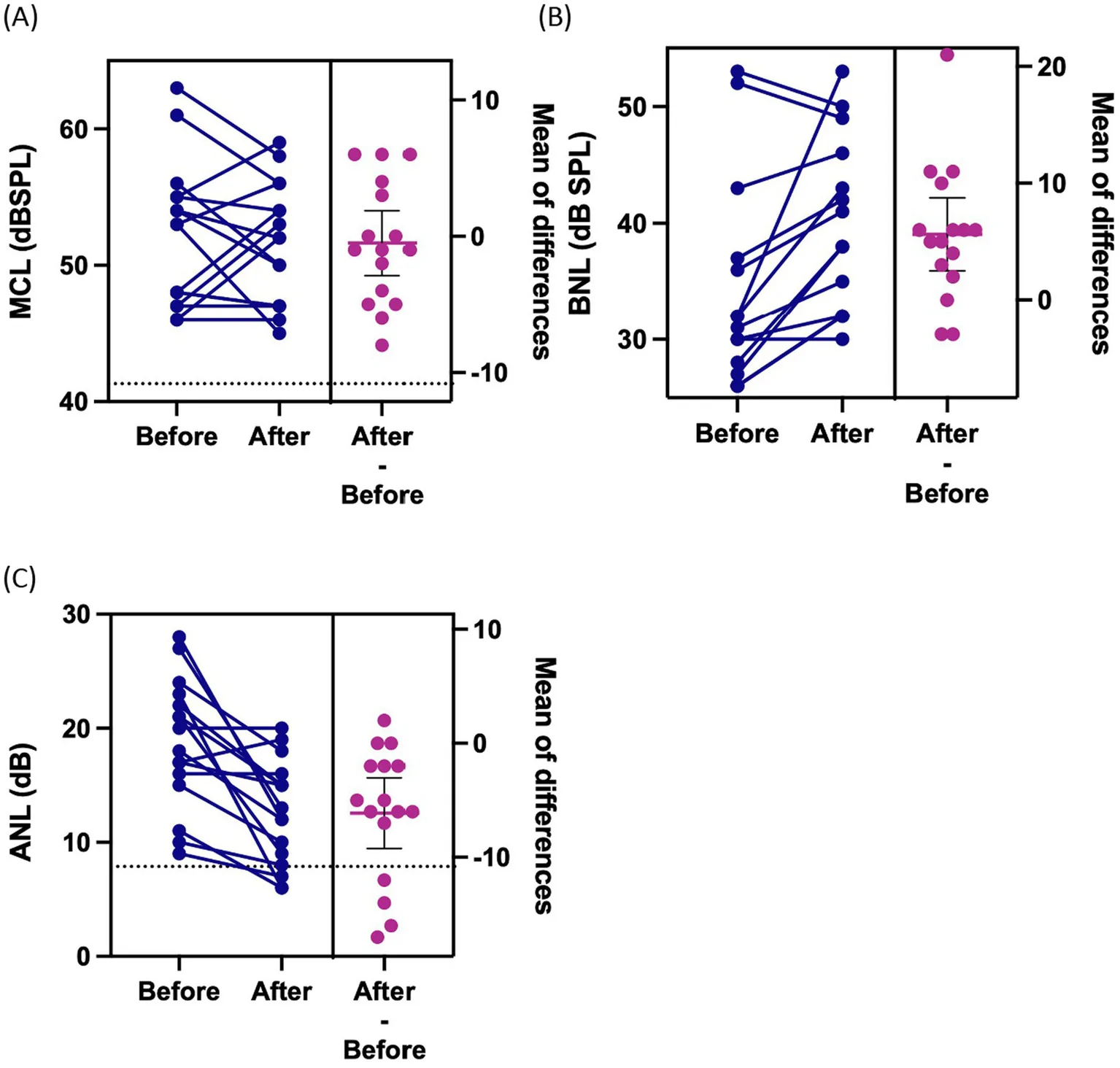
Estimation plots. **(A)** The MCL. **(B)** BNL. **(C)** ANL for each participant before and after auditory training (blue) and individual differences (purple). The purple lines indicate the mean differences ± the standard deviation. Dotted lines meaningful change boundary.

No significant change was observed for MCL, *t*(15) = 0.45, *p* = 0.662 (Figure 2), indicating that the preferred listening level for speech remained stable across the intervention period. In contrast, BNL increased significantly following training, *t*(15) = 3.83, *p* = 0.0016. Mean BNL increased from 33.4 dB (SD = 8.81) at baseline to 39.1 dB (SD = 7.48) post-training, representing an average increase of 5.63 dB (SD = 5.88). Together, the increase in BNL in the absence of any change in MCL indicates that participants became more willing to tolerate competing background noise while listening to speech after undertaking AOIL training.

### 3.5 Noise sensitivity

Perceived noise sensitivity, as measured by the NoiSeQ, decreased significantly following auditory training. Mean NoiSeQ scores decreased from 144.4 (SD = 17.0) before training to 133.6 (SD = 17.5) after training (Figure 3) suggesting reduced perceived noise sensitivity following the intervention. A paired-samples *t*-test demonstrated a significant difference between pre- and post-training scores, *t*(15) = 2.87, *p* = 0.0116. Mean NoiSeQ scores decreased by 10.81 points (SD = 15.06), from 144.4 (SD = 16.99) before training to 133.6 (SD = 17.45) after training. There was an average improvement of approximately 11 points following AOIL training. The standard error of the mean difference was 3.77, and the 95% confidence interval (CI) for the mean difference ranged from −18.84 to −2.79. Because the confidence interval does not include zero, these findings provide additional evidence that a true change occurred following the intervention. The magnitude of the effect was moderate-to-large. The partial eta-squared value (η^2^p = 0.36) indicates that approximately 36% of the variance in scores was attributable to the intervention effect, representing a substantial within-participant change.

**Figure 3:**
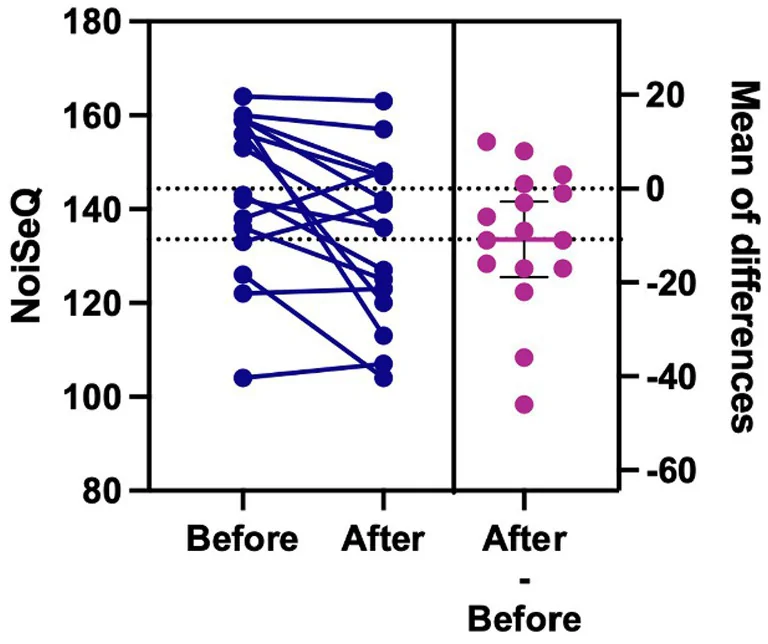
Estimation plot of NoiSeQ scores for each participant before and after auditory training (blue) and individual differences (purple). The purple lines indicate the mean differences ± the standard deviation. Dotted lines meaningful change boundaries.

These findings indicate that participants perceived themselves as less sensitive to environmental noise following completion of the AOIL intervention.

### 3.6 Correlations and co-morbidities

An exploratory correlation analysis (*p* ≤ 0.05, reported) revealed that age was positively associated with training adherence (*r* = 0.63) and negatively associated with LDL improvement across multiple frequencies (*r* = −0.49 to −0.68), indicating that younger participants generally demonstrated greater gains in loudness tolerance. Baseline hyperacusis severity (IHS score) was not significantly associated with any outcome measure. Training adherence was not significantly correlated with changes in ANL, NoiSeQ, or LDL measures. A moderate negative association was observed between improvements in ANL and reductions in NoiSeQ scores (*r* = −0.49), suggesting convergence between objective and subjective measures of sound tolerance. Changes in LDL were strongly correlated across frequencies and ears, indicating that improvements in loudness tolerance tended to occur broadly rather than being frequency-specific. The study was not powered to differentiate the effects of gender or hearing loss. There were 11 participants with some degree of tinnitus and five participants without. For descriptive purposes the “with tinnitus” group had smaller NoiSeQ changes (9.36, SD = 13.75) than the non-tinnitus group (14.0, SD = 18.96), the sample is too small for a meaningful statistical comparison.

## 4 Discussion

This study investigated whether Auditory Object Identification and Localisation training could improve sound tolerance, acceptance of background noise, and perceived noise sensitivity in individuals with hyperacusis. This was a preliminary study to provide proof-of-concept for the training used. Across all primary outcome measures, significant improvements were observed following 2 weeks of training. Specifically, participants demonstrated increased LDLs, reduced ANLs, and lower NoiSeQ scores after completing the intervention. Taken together, these findings suggest that AOIL training may positively influence several clinically relevant dimensions of hyperacusis. Importantly, the observed improvements were achieved using a brief, home-based, digital intervention, highlighting the potential utility of auditory training as an accessible management option for individuals with decreased sound tolerance.

### 4.1 Effects of AOIL training on sound tolerance

The most notable finding was the significant increase in LDLs following AOIL training. LDLs are widely used in the assessment and monitoring of hyperacusis and represent a behavioural measure of an individual’s tolerance to sound. The approximately 10 dB improvement observed across frequencies suggests that participants were able to tolerate higher sound levels following training. There was not a control group to comparison against, bur this finding differs from previous AOIL studies conducted in individuals with normal sound tolerance (Bees et al., 2019), which reported no significant changes in LDLs following training. In those studies, baseline LDLs were already within normal limits, leaving limited opportunity for improvement. By contrast, participants in the present study demonstrated LDLs characteristic of hyperacusis prior to training, indicating clinically reduced sound tolerance. The improvement observed therefore suggests that AOIL training may be particularly beneficial when sound tolerance is abnormally reduced. Bourez et al. (2025) identified that hyperacusis participants had an average of 13 dB lower LDLs than controls. If the present results were generalised, it would suggest LDLs approaching normal perception post-training (Bourez et al., 2025). The present findings are also broadly consistent with previous intervention studies demonstrating improvements in LDLs following sound therapy, desensitisation programmes, and tinnitus retraining therapy (Formby and Gold, 2002; Vidal et al., 2022). Collectively, these studies suggest that loudness tolerance is modifiable and that mechanisms underlying hyperacusis can be altered through targeted intervention. Improvements were observed across all tested frequencies, with no significant frequency effects. This suggests that hyperacusis-related loudness intolerance was affected similarly across the frequency range examined. These results support the view that hyperacusis in our participants reflected a generalised alteration in sound tolerance rather than a frequency-specific disturbance. Similarly, no significant differences between ears were observed either before or after training. According to conventional benchmarks, the change in LDL shoed a large effect size which suggests that the change observed was not only statistically significant but also potentially clinically meaningful. Notably, the effect size for ANL improvement was larger than that observed for NoiSeQ outcomes, indicating that AOIL training may have had a particularly strong influence on behavioural measures of sound acceptance.

### 4.2 Effects of AOIL training on noise acceptance

A second major finding was the significant reduction in ANLs following auditory training. ANLs provide a measure of the amount of background noise an individual is willing to accept while listening to speech and therefore offer insight into real-world listening tolerance (Nabelek et al., 2004). The reduction in ANLs observed in this study indicates that participants became more accepting of competing background noise following training. These findings extend previous AOIL research in individuals with normal hearing and normal sound tolerance, where improvements in ANLs have also been reported (Bees et al., 2019). The current study demonstrates for the first time that similar improvements can be achieved in individuals with hyperacusis. Improving acceptance of background noise may have practical implications for communication, work, social participation, and everyday activities.

Analysis of the ANL components provides additional insight into the nature of these improvements. Most Comfortable Levels (MCLs) remained unchanged following training, suggesting that preferred listening levels were stable. In contrast, Background Noise Levels (BNLs) increased significantly after training. Therefore, the reduction in ANLs were driven by an increased willingness to accept competing noise rather than changes in preferred speech levels. This pattern mirrors findings from previous AOIL studies (Bees et al., 2019) and is consistent with current understanding of ANL mechanisms. Research suggests that BNLs, and by extension ANLs, are influenced by higher-order auditory processes (Bees et al., 2019). The selective improvement in BNLs therefore aligns closely with the intended targets of the AOIL intervention.

### 4.3 Effects of AOIL training on noise sensitivity

The study also demonstrated a significant reduction in NoiSeQ scores following training, indicating decreased self-perceived noise sensitivity across a range of everyday listening situations. The current findings suggest that individuals’ subjective reactions to environmental sounds can be modified through auditory training. Noise sensitivity has been conceptualised as a relatively stable personality characteristic. However, more recent models propose that noise sensitivity reflects a dynamic interaction between auditory processing, attention, emotional responses, arousal, and contextual factors (Welch et al., 2022). The improvements observed in the present study are more consistent with this model than with the notion of noise sensitivity as a fixed trait. Participants reported reduced disturbance from environmental noise following intervention, suggesting that aspects of noise sensitivity may be amenable to rehabilitation. It remains unclear whether improvements in NoiSeQ scores were secondary to improvements in sound tolerance and noise acceptance or whether noise sensitivity represents a distinct construct that was independently influenced by the intervention. Previous research has generally reported weak associations between LDLs and noise sensitivity measures, suggesting that these assessments capture different aspects of auditory experience (Jahn, 2022). Consequently, AOIL training may have affected multiple related but distinct dimensions of hyperacusis.

### 4.4 Relationships between findings

An exploratory correlation analysis provided some insight into factors that may influence treatment response, although these findings should be interpreted cautiously given the small sample size. Age was positively associated with training adherence, suggesting that older participants tended to complete a greater proportion of the prescribed training sessions. However, age was also negatively associated with improvements in LDL across multiple frequencies, indicating that younger participants generally demonstrated larger gains in loudness tolerance. This pattern may reflect age-related differences in auditory plasticity, attentional processes, or responsiveness to perceptual training, although the present study was not designed to evaluate these mechanisms. Baseline hyperacusis severity, as measured by the IHS, was not significantly associated with any outcome measure, suggesting that participants experienced benefit across a range of initial symptom severity levels. Training adherence was not significantly correlated with changes in ANL, NoiSeQ, or LDL outcomes, providing no clear evidence of a dose–response relationship within the range of adherence observed in this cohort. A moderate association between improvements in ANL and reductions in NoiSeQ scores suggests some convergence between behavioural and self-reported measures of sound tolerance. In contrast, the strong correlations observed among LDL changes across frequencies and ears indicate that improvements in loudness tolerance tended to occur broadly rather than being frequency-specific.

The present study was not powered to evaluate the potential influence of participant characteristics, including sex, hearing status, or co-occurring auditory conditions. Eleven participants reported tinnitus while five did not. Although no meaningful statistical comparison was possible, descriptive analyses showed that participants with tinnitus demonstrated smaller average reductions in NoiSeQ scores (mean = 9.36, SD = 13.75) than participants without tinnitus (mean = 14.0, SD = 18.96). Given the recognised overlap between tinnitus and hyperacusis, as well as the possibility that other sound sensitivity phenotypes may influence treatment response, future studies should recruit larger samples that allow these variables to be evaluated as potential moderators of intervention effectiveness (Vanneste et al., 2026).

### 4.5 Potential mechanisms

Although the present study was not designed to directly investigate mechanisms, several observations provide clues regarding how AOIL training may have produced the observed benefits. The AOIL was specifically developed to target auditory object identification, localisation, and selective attention. Throughout training, participants were required to focus on relevant sounds, distinguish them from competing auditory information, and identify their spatial locations. Such tasks are designed to directly engage auditory scene analysis and alter attentional control processes (Searchfield et al., 2007). The significant improvements observed in ANLs and noise sensitivity are particularly consistent with this explanation. Improvement in attention may enable individuals with hyperacusis to more effectively filter, organise, and interpret auditory information, reducing the impact of environmental sounds. The mechanism underlying improvements in LDLs is less certain. It is possible that repeated exposure to a wide range of sounds during training produced an element of controlled auditory desensitisation. Alternatively, improvements in attentional allocation and auditory object processing may have altered the way sounds were perceived relative to a reference norm [adaptation level, (Helson, 1948)] resulting in increased tolerance. These explanations are not mutually exclusive, and both processes may have contributed to the observed changes. The effectiveness of AOIL training highlights the potential importance of auditory attention and auditory scene analysis in the experience of decreased sound tolerance.

### 4.6 Clinical implications

The present findings have several important clinical implications. The results provide evidence that sound tolerance, background-noise acceptance, and perceived noise sensitivity are modifiable in individuals with hyperacusis. This is an encouraging finding for both clinicians and patients, as hyperacusis is often perceived as difficult to treat and associated with substantial functional limitations. Interventions targeting auditory attention and auditory scene analysis may represent a useful addition to existing management approaches. Current treatments for hyperacusis frequently focus on sound desensitisation, counselling, and management of emotional distress. AOIL training introduces an alternative approach by actively targeting higher-order auditory processing. The intervention demonstrated strong feasibility. Most participants completed the prescribed training schedule, and engagement levels were generally high. Also, this study introduces ANLs as a potentially valuable outcome measure in hyperacusis research and clinical practise. The significant changes observed suggest that ANLs may provide useful information about listening difficulties in everyday environments and may be sensitive to treatment-related change.

### 4.7 Strengths and limitations

A key strength of this study was the use of multiple outcome measures capturing different dimensions of hyperacusis, including LDLs, ANLs, and perceived noise sensitivity (NoiSeQ). This enabled a broader assessment of treatment effects than studies relying on a single measure. Participant selection was also strengthened by the use of the IHS to identify individuals with clinically significant hyperacusis symptoms. The sample included a range of ages and symptom severities, increasing clinical relevance. However, several limitations should be acknowledged. The study included a relatively small sample and lacked a control group. Consequently, causal conclusions regarding treatment effectiveness should be made cautiously. Placebo effects, expectation effects, or natural symptom fluctuations cannot be excluded.

The study also relied primarily on behavioural and self-report measures. Objective neurophysiological measures were not collected, limiting conclusions regarding underlying mechanisms. Future investigations incorporating electroencephalography (EEG) or other objective assessments may provide additional insight into the processes affected by AOIL training. Finally, participants presented with diverse histories of hyperacusis, comorbidities, and suspected causes. While this reflects real-world clinical populations, future studies may benefit from examining more specific subgroups, including individuals with different hyperacusis types, autism spectrum disorder, traumatic brain injury, or other associated conditions. Although the sample reflects the diversity commonly seen in clinical hyperacusis populations, participants varied considerably in age, hearing status, sex, and the presence of potentially relevant co-occurring auditory and sound sensitivity conditions. The modest sample size precluded meaningful subgroup analyses or the inclusion of multiple covariates within statistical models. Consequently, it was not possible to determine whether factors such as tinnitus, misophonia, hearing loss, age, or sex influenced treatment outcomes. These variables may have contributed to the observed variability in response and should be considered potential confounders when interpreting the findings.

The findings should also be interpreted in the context of the declared conflict of interest, as the senior author was involved in the development of the QCore™ platform. While this does not affect the reported data, the combination of a preliminary single-arm design, absence of blinding, and investigator involvement in the intervention under evaluation increases the potential for conscious or unconscious interpretive bias. Consequently, independent replication using controlled and blinded methodologies is warranted.

### 4.8 Future directions

Future research should evaluate AOIL training using larger, controlled study designs. Longer follow-up periods are also required to determine whether improvements are maintained after training cessation. Studies incorporating objective measures of auditory processing, attention, working memory, sensory gating, and cortical activity may help clarify the mechanisms underlying treatment-related changes. Studies incorporating extended high-frequency audiometry and additional measures of cochlear integrity (e.g., otoacoustic emissions) would help to better characterise auditory status within hyperacusis cohorts. Such investigations could determine whether improvements in LDLs, ANLs, and noise sensitivity arise from shared or distinct cochlear-neural processes. Future research should seek to address participant heterogeneity. Studies may recruit more homogeneous cohorts with respect to clinical presentation and auditory characteristics, or alternatively, recruit substantially larger samples that permit appropriate statistical control of important co-existing variables. In particular, future investigations should examine the influence of tinnitus, misophonia and other sound hypersensitivity phenotypes, hearing loss, age, and sex on treatment outcomes. Phenotype-specific screening (Lopez-Escamez et al., 2026) and subgroup analyses would help determine whether specific subgroups derive greater benefit from AOIL training and would improve understanding of the factors that moderate intervention effectiveness.

## 5 Conclusion

A two-week AOIL auditory training programme demonstrated an association between training and improvements in in sound tolerance, acceptance of background noise, and perceived noise sensitivity in individuals with hyperacusis. Improvements were reflected by increased LDLs, reduced ANLs, higher BNLs, and lower NoiSeQ scores following training. The findings support the potential role of auditory attention and auditory scene analysis in hyperacusis and suggest that interventions targeting these processes may provide clinically meaningful benefits. While further controlled research is required to confirm efficacy and clarify mechanisms, AOIL training represents a promising, accessible, and engaging approach to hyperacusis management.

## Data Availability

The raw data supporting the conclusions of this article will be made available by the authors, without undue reservation.

## Footnotes

1
https://www.ucsfhealth.org/care/services/hyperacusis-care
